# *Para Niños Saludables*: A Community Intervention Trial to Reduce Organophosphate Pesticide Exposure in Children of Farmworkers

**DOI:** 10.1289/ehp.10882

**Published:** 2008-01-22

**Authors:** Beti Thompson, Gloria D. Coronado, Eric M. Vigoren, William C. Griffith, Richard A. Fenske, John C. Kissel, Jeffry H. Shirai, Elaine M. Faustman

**Affiliations:** 1 Cancer Prevention Program, Fred Hutchinson Cancer Research Center, Seattle, Washington, USA; 2 Department of Health Services; 3 Department of Epidemiology; 4 Department of Environmental and Occupational Health Sciences, School of Public Health and Community Medicine, University of Washington, Seattle, Washington, USA

**Keywords:** children, community intervention, farmworkers, organophosphate pesticides, pesticide exposure, randomized trial, rural community

## Abstract

**Background:**

Exposure to organophosphate (OP) pesticides is an occupational hazard for farmworkers and affects their children through the take-home pathway.

**Objectives:**

We examined the effectiveness of a randomized community intervention to reduce pesticide exposure among farmworkers and their children.

**Methods:**

We conducted a baseline survey of a cross-sectional sample of farmworkers (year 1) in 24 participating communities. Communities were randomized to intervention or control. After 2 years of intervention, a new cross-sectional survey of farmworkers was conducted (year 4). Farmworkers with a child 2–6 years of age were asked to participate in a substudy in which urine was collected from the farmworker and child, and dust was collected from the home and the vehicle driven to work.

**Results:**

The median concentration of urinary metabolites was higher in year 4 than in year 1 for dimethylthiophosphate (DMTP) and dimethyldithiophosphate in adults and for DMTP for children. There were significant increases within both the intervention and control communities between year 1 and year 4 (*p* < 0.005); however, the differences were not significant between study communities after adjusting for year (*p* = 0.21). The dust residue data showed azinphos-methyl having the highest percentage of detects in vehicles (86% and 84% in years 1 and 4, respectively) and in house dust (85% and 83% in years 1 and 4, respectively). There were no significant differences between intervention and control communities after adjusting for year (*p* = 0.49).

**Conclusions:**

We found no significant decreases in urinary pesticide metabolite concentrations or in pesticide residue concentrations in house and vehicle dust from intervention community households compared with control community households after adjusting for baseline. These negative findings may have implications for future community-wide interventions.

Organophosphate (OP) pesticides are ubiquitous insecticides in use in U.S. agriculture [U.S. Environmental Protection Agency (EPA) 2004]. Exposure to such pesticides is a significant occupational health hazard for farmworkers (For Healthy Kids 1999a, 1999b; Villarejo 2003; Villarejo and Baron 1999) because OP pesticides are associated with harmful health effects in adults. Further, adult farmworkers are thought to take the pesticides home on their boots, clothing, and skin. This is of great concern because children of farmworkers are thought to be exposed to pesticides largely through the take-home pathway (Eskenazi et al. 1999; Faustman et al. 2000; Flocks et al. 2001; Lu et al. 2000; McCauley et al. 2006b; Simcox et al. 1995; Thompson et al. 2003). Pesticide residue on farmworkers’ clothing, shoes, and skin is brought into the home from the fields, and these residues then persist in the indoor environment where they are a potential source of exposure to farmworkers’ families.

An increasing body of scientific literature has shown that children have a greater vulnerability to the effects of chemical exposures than do adults. This can be attributable to a variety of factors including development of sensitive organ systems (i.e., brain, immune, respiratory) as well as different capacity to metabolize and eliminate compounds than adults (Anderson et al. 2000; Faustman et al. 2000; International Programme on Chemical Safety 2007; National Research Council 2000). For example, children can have less-developed metabolic systems than adults and can break down pesticides at slower rates (Eskenazi et al. 1999; Faustman et al. 2000). Further, children can experience greater environmental exposures by engaging in hand-to-mouth behaviors that increase their risk of ingesting pesticides found in the home (McCauley et al. 2006b; Quandt et al. 2004). Studies of children have identified possible risks for the development of cancers, birth defects, and abnormal reflexes, as well as neurologic impairments after environmental exposures (Blain 2001; Guillette et al. 1998; Kirkhorn and Schenker 2002; Mills and Yang 2003; Rohlman et al. 2005; U.S. General Accounting Office 2000; Young et al. 2005). Understanding and determining what exposures are harmful and how to intervene and reduce exposures is essential. The social implications of such neurologic impairments can be far reaching; a recent report demonstrates that a five-point reduction in IQ levels in children corresponds to a 50% reduction in the number of geniuses and a 66% rise in the number of children who need specialized education or health care services (Greater Boston Physicians for Social Responsibility 2000). The U.S. EPA uses uncertainty factors to protect children from environmental risks when data are uncertain. Identification of new, relevant data for children’s exposure and their response to pesticides is needed to inform our risk assessment and replace some uncertainty factors. Recent data from Furlong et al. (2006) suggest that children may be much more susceptible to the effects of pesticides than adults (based on paraoxonase 1 levels in the blood). Hence, additional studies of how, when, and to what pesticides children may be exposed are critical.

In this project we used a community-based participatory research strategy. There is growing recognition that the determinants of pesticide exposure extend beyond individual factors to the community in which the individual lives. Community infrastructure, such as spraying practices, farmworker protection, and social norms governing pesticide protection, can influence exposure levels. Further, a limited number of previous investigations have designed programs that involve communities in efforts to reduce pesticide exposure among high-risk groups (Arcury et al. 1999, 2000, 2001; Quandt et al. 2001a, 2001b). Moreover, few previous studies have used environmental or biomarker data to demonstrate influences of a given intervention on exposure levels.

Using data from cross-sectional samples of community residents in 24 communities in an agricultural area of eastern Washington State, we examined the effectiveness of a community-wide intervention, via a community randomized study, to reduce pesticide exposure among farmworkers and their children. The major hypothesis was that a community-based intervention trial would result in lower pesticide exposure among farmworkers and their children, compared with a control group with no intervention. Our primary outcomes were farmworkers’ and children’s urinary metabolite levels and pesticide residue levels in dust found in the home and in the vehicle driven to work. In this report, we examine the environmental and biomarkers evidence to identify differences in pre- and postintervention levels of exposure.

## Methods

### Setting

This trial was conducted in the Yakima Valley of Washington State. A description of the area has been given in detail elsewhere (Thompson et al. 2003). Briefly, the Yakima Valley is a major agricultural area, heavily populated by Hispanics, who do much of the agricultural work. In the Yakima Valley, a region that includes many small agricultural communities, the percentage of Hispanics is estimated at > 50% (Project Health Community Board, personal communication; U.S. Census Bureau 2002). Apples, pears, peaches, cherries, grapes, and hops are the primary crops (U.S. Department of Agriculture 2002a). Many members of the Hispanic population are involved in agricultural work, specifically in harvesting, pruning, thinning, and other care of the many crops grown in the Lower Yakima Valley. The pesticides used on the fruit group include organophosphate pesticides such as azinphos-methyl, phosmet, methyl-parathion, among others (U.S. Department of Agriculture 2002b).

The valley is approximately 150 miles long and 75 miles wide. There are approximately 20 small towns (size ranges from 300 to 11,000) and a number of labor housing clusters in the valley. Because community and labor camps were randomized for participation in the study, there was a chance that contamination could occur. Contamination was monitored by including questions in the final survey that asked about awareness of and participation in intervention activities.

### Study design

The overall design of the study has been described in detail elsewhere (Curl et al. 2002; Thompson et al. 2003). Essentially, 24 communities were randomized to an intervention or control condition. After developing a community advisory board (CAB), as described below, we conducted a baseline survey of a cross-sectional sample of farmworkers (year 1). Key informants in the communities, including CAB members, provided information regarding localities within communities where farmworkers were thought to live. Within those communities, we formed a list of addresses by driving around the designated areas. An interviewer then went to each address and ascertained whether a farmworker lived in the residence. If a farmworker was present, she or he was interviewed for the baseline assessment.

After baseline assessment, communities were blocked into pairs based on community size and percent Hispanic in the community, and treatment arm of another ongoing study (to control for general behavioral changes that might occur as a result of being involved in an intervention community in that project), and then randomized within a pair to intervention or control status. A total of 16 communities and eight labor camps were randomized to intervention or control.

After 2 years of intervention, a new cross-sectional survey of farmworkers was conducted in the 24 participating communities (year 4). The major trial outcomes include *a*) differences between intervention and control communities in urinary organophosphate metabolites of children 2–6 years of age who reside with farmworkers (primary); *b*) differences in urinary organophosphate metabolites of farm-workers (secondary); *c*) differences in house dust and vehicle dust in the environments of the farm workers (secondary); and *d*) differences in self-reported knowledge, attitudes, and practices of farmworkers regarding protection of their children from pesticide exposure (secondary). Here we report on the urinary metabolites and dust data.

### Intervention

#### Community organization

The overarching theoretical framework for the study was a community organization approach (Bracht et al. 1998; Israel et al. 1998; Thompson et al. 2001). A community organization approach requires that a group affected by a problem be involved in finding solutions to the problem (Minkler 1998; Thompson and Kinne 1999). Toward that end, an initial community analysis led to the formation of a CAB. The process has been described in detail elsewhere (Thompson et al. 2001). Briefly, qualitative research was used to identify relevant individuals and groups that were involved in some fashion in pesticide issues in the Yakima Valley. A planning committee (PC) nominated and recruited individuals to serve on the CAB. CAB representation consisted of 18 individuals from 16 organizations including farm-workers, growers and their associations, regulatory agencies, health department, the U.S. Department of Agriculture, the U.S. Department of Labor and Industries, the local Region X EPA, local media including the Spanish-speaking radio station, the farm-worker union, local farmworkers clinics, and farmworker advocates. The CAB members came from many different towns in the valley.

Once formed, the CAB became a full-scale partner of the project. It was responsible for hiring staff to work in the valley, contributed to the research design, and recommended a number of intervention strategies. From a research design perspective, the CAB recommended that dust be collected from vehicles as well as houses, arguing that dust and pesticide residues from vehicles were tracked into the home. From an intervention viewpoint, the CAB encouraged intervention activities in preschool programs to reach farmworkers who had small children. Throughout the project, the CAB made recommendations on how and when the data collected from the project should be conveyed to the community, the news media, and peer-reviewed journals. In short, the CAB was heavily involved in all aspects of the project. CAB members were aware of randomization and were given information on the randomization outcome. CAB members were enthusiastic about the project and saw it as an opportunity to answer important, unknown questions about pesticide exposure.

#### Intervention components

A 2-year, comprehensive intervention plan included intervention activities at the community, organizational, small group, and individual levels. Intervention components were based on the extant literature and recommendations of the CAB. At the community level, health fairs, community festivals, and block parties were held. Local media spread messages about the project and pesticide protection. Health fairs are a common event in the valley, and the project formed a road-ready booth that was erected and staffed at intervention community health fairs. The same booth was used at community festivals and at block parties. Staffers of the booth provided information on the risks of pesticides for children, symptoms of pesticide exposure, information on protecting oneself and one’s children from pesticides, and an overview of the project. Children’s coloring books, balloons, and other artifacts also spread the pesticide messages. A pesticide puppet show, developed by a local university, was shown at block parties and festivals. The puppet show emphasized the importance of avoiding fields where pesticides were used and ways that children could protect themselves from pesticides.

At the organizational level, several groups were targeted for pesticide messages. These included elementary schools, where a calendar contest was held annually to promote pesticide protection messages; churches, where infants were provided a package containing pesticide protection messages and a bib reading “Keep me pesticide free,” and where after-mass coffees promoted the pesticide protection messages; classes in English as a second language and citizenship, where messages about protecting one’s family from pesticides were included in the curriculum; and preschools such as Head Start, where a preschool curriculum was developed and taught in all intervention community preschools. Other organizational venues were worksites including orchards and farms, farm-worker clinics, and the farmworker union. In many organizations, group discussions were held on the dangers of pesticides, especially for children. Sample packets of detergent, clothes sorting bags, bins for storing boots outside, and shower kits were distributed in organizations. Individuals were instructed on ways to keep pesticides from being tracked into the home, including instructions on cleaning the home and vacuuming the car regularly.

At the small-group level, lay health educators (*promotoras*) were used to spread messages about pesticides. A popular small-group-level activity was home health parties. A home health party was a small gathering of friends and relatives in the home of a “host” or person who agreed to hold the party. Typically, a trained *promotora* gave a guided 30- to 45-min discussion about a specific pesticide topic. The *promotora* used simple charts and props to give information about ways to reduce pesticide risks. The small-group format fostered discussion and opportunities to obtain more information. In the 2-year intervention period, > 1,100 home health parties were held.

At the individual level, volunteer *promotoras* as well as staff talked to individuals about protecting one’s family from pesticide exposure. A volunteer training handbook was developed. Volunteers went door to door and spoke at grocery stores and other places frequented by farmworkers. They also distributed laundry kits, shower kits, and other samples created by the project.

### Baseline (year 1) and final assessment (year 4)

From randomly selected households, all adult agricultural workers were identified and their first names and birth dates listed on a questionnaire roster. From each household, one farmworker was selected (based on the adult with the first birthday after 1 April) to complete an in-person interview. Farmworkers with a child 2–6 years of age in the household were asked to participate in a part of the study in which urine was collected from the farm-worker and eligible child, dust was collected from the home, and dust was collected from the vehicle driven to work.

All respondents were asked to give verbal consent to participate; they were given a $5 coupon to a local grocery store as an incentive. Adult participants who agreed to take part in the urine and dust collection were given $50, and the participating child was given a small stuffed toy. For this part of the study, adult respondents signed informed consent. The study protocol and data collection procedures were reviewed and approved by the Human Subjects Review Board at the University of Washington (No. 98-6567-C) and the Institutional Review Board at the Fred Hutchinson Cancer Research Center (No. 5101). The study complied with all applicable requirements of the United States. All human participants gave written informed consent before participating in the study.

In addition to the above, we conducted extensive process evaluation to help ascertain “dose”—the extent to which intervention activities were conducted in various communities. Final interview questionnaires also contained information on awareness of and participation in intervention activities to help assess contamination between intervention and control communities.

#### Interviewing

In-person interviews were conducted by 22 locally-hired and trained bilingual interviewers. Three 6-hr training sessions were conducted by bilingual project staff. The training addressed strategies for approaching households, methods for asking questions in a standard manner, methods of editing questionnaires, and rules for documenting household contacts and survey dispositions. Interviewers were tested and certified. Interviewing for the baseline survey took place between 1 June 1999 and 15 October 1999, and for the final survey between 1 June 2002 and 30 September 2002.

#### Interview instrument

The interview contained 87 items that included questions about agricultural tasks, general pesticide exposure in job tasks, farmworker protective practices at work, employer practices at work, family protective practices, and demographics. In addition, questions asked about awareness of and participation in project activities.

To assess agricultural job tasks and pesticide exposure, we gave respondents a list of agricultural job tasks (e.g., harvesting, pruning, thinning, mixing pesticides, applying pesticides) and asked which tasks they had performed in the preceding 3 months. For each job task answered in the affirmative, respondents were asked whether they had come into contact with pesticides while performing the task (yes/no). They also were asked, in general, how frequently, when working, pesticides touched their clothing, touched their skin, they breathed in pesticide dust or chemical fumes, and they were dusted or sprayed with pesticides (almost every day, once in a while, rarely, or never).

To assess employer protective practices at work, we asked respondents about the presence of worksite facilities required by the Occupational Safety and Health Administration and the worker protection standards (WPS) of the U. S. EPA (U.S. EPA 1992, 1994). The WPS regulation is aimed at reducing the risk of pesticide poisonings and injuries among agricultural workers and pesticide handlers. Employers must provide drinking water, bathrooms, water for washing hands, soap for washing hands, towels for drying hands, eyewash stations (water for flushing eyes), and showers (these latter are required only for pesticide handlers) for all workers who enter any field in which pesticides have been used. Response categories for the presence of such facilities included “always, sometimes, rarely, or never.”

We assessed home protective practices by farmworkers by questions developed from studies with farmworkers conducted by the National Institute for Occupational Safety and Health (Cameron L, Lalich N, Coronado G, unpublished data). Questions asked respondents to report whether they washed hands immediately after work, took off boots immediately after returning home from work, washed work clothes separately from the rest of the family laundry, and held or hugged their children before changing from work clothes. Response categories were “always, usually, sometimes, rarely, or never.” Farmworkers were also asked how soon after returning home from work they removed their work clothes, and showered or bathed (< 1 hr, 1–2 hr, > 2 hr). The desirable response was < 1 hour. This was thought to account for time spent driving home, picking up children from child care, and the like.

Most of the sociodemographic variables were assessed by 2000 Census questions. Sex was determined by self-report. Age was ascertained from the rostering information that obtained birth day, month, and year for each adult member of the household. Respondents could state how many years of education they had received; these were later collapsed into 4th grade or less, 5th through 8th grade, 9th through 12th grade (no diploma), high school graduate or more. Ethnicity was self-reported and included white, Hispanic, African American, American Native, Eskimo or Aleut, Asian or Pacific Islander, and other. Marital status was self-reported as married (or living as married), widowed, divorced/separated, never married. Annual household income was self-reported as < $10,000, $10,001–15,000, $15,001–25,000, or > $25,000. Respondents self-reported the number of children (< 18 years of age) in the household.

### Biomarker and dust data

A subsample of farmworkers had a child 2–6 years of age residing in their households, and consented to participate in the urine and dust collection portion of the study. Sampling protocols were based on standard operating procedures developed at the University of Washington and are reported in detail by Curl et al. (2002). For urine, we collected two or three independent voids separated by a minimum of 3 days and a maximum of 2 weeks from one child and one farmworker in each eligible household. Samples were refrigerated immediately after being produced and transported to the field laboratory in coolers. In the laboratory, samples were refrigerated until all samples for a participant had been provided. At that point, equal volumes of the independent urine samples (approximately 15 mL each) were pooled for each individual, then small tubes of pooled urine were drawn, frozen, and shipped to the laboratory for analysis. Our bilingual urine collectors attended three 6-hr training sessions. The training addressed the importance of obtaining sufficient urine, of refrigerating the urine immediately after the sample was provided, of the timeline for sample collection, rules for documenting household contacts, and form dispositions. Sample collectors were tested and certified. Urine samples were stored at –10°C, shipped on ice to the laboratory at the University of Washington, and again stored at –10°C. Urine samples were analyzed at baseline (year 1) and at the end of the study (year 4) for OP metabolites according the method described by (Moate et al. 1999). The analyzed OP metabolites included dimethylphosphate (DMP), dimethylthiophospate (DMTP), and dimethyldithiophosphate (DMDTP), which corresponded to the pesticides most commonly used in the valley.

Using a Nilfisk vacuum cleaner (Nilfisk of America, Malvern, PA), house dust was collected from the residences of the farmworkers. A cleaned vacuum and fresh polyliner bag, along with a clean vacuum hose and wand, were used for each household. Procedures for house and vehicle dust sampling were also developed by the University of Washington (Curl et al. 2002). Areas were vacuumed in a standardized manner. A square half-meter by half-meter template was used as a guide. Depending on flooring type, 4–8 templates were vacuumed. The area vacuumed was where the parent reported “the child played most frequently.” After dust collection, the vacuum bag and polyliner were removed and placed in a plastic bag and stored at –10°C for transfer to the laboratory at the University of Washington for analysis. Vehicle dust was collected in a similar manner. The footwells, front and rear (except for trucks without rear footwells), of the vehicle were thoroughly vacuumed. After dust collection, the vacuum bag and polyliner were removed and placed in a plastic bag and stored at –10°C for transfer to the laboratory at the University of Washington for analysis. Dust samples were analyzed for OP residues according to the procedures described by Moate et al. (2002), including azinphos-methyl, phosmet, and malathion, the pesticides in most common use in the valley.

### Analysis

The percentages of detectable OP metabolites are based on the number of urine samples analyzed. The total number of 218 households is reduced by the number of missing samples and, in the case of the dust samples, by the number of samples with insufficient mass for analysis.

The analysis of the 218 households that provided urine and dust samples employed a hierarchical Bayesian model evaluated in the Markov chain Monte Carlo (MCMC) software WinBUGS (Spiegelhalter et al. 2003), which allowed unbiased analysis of the censored and missing data (Vigoren EM, Griffith WC, Faustman EM, unpublished data). The log-transformed data were modeled as multivariate normal distributions with conjugate, noninformative priors. Each set of four community categories (intervention year 4/year 1, control year 4/year 1) was given its own distribution and covariance matrix. The geometric mean and geometric standard deviations were calculated from the posterior mean and variance distribution parameters from these models, using 500,000 MCMC iterations after a burn-in of 25,000 iterations.

The magnitude and significance of differences between year 4 and year 1 were summarized in the ratio (fold difference) of the geometric means (GM) and the associated *p*-values. The ratio of these ratios for the intervention and control groups identifies the impact of intervention after adjusting for the year effect. The probability (*p*-value) that one GM was greater than another was the fraction of the MCMC iterations in which the first GM exceeded the other. For clarity, because a *p*-value of 0.95 is just as extreme as a value of 0.05, the *p*-values were all transformed to the range 0–0.5 with the function min[*p* (*x* > *y*), *p* (*x* < *y*)].

## Results

### Response rates

For the baseline assessment, we identified 1,263 addresses. Of these, 468 had no farmworkers, 114 were vacant households, 23 had nobody home after five visits, 26 households refused to participate, and 46 were ineligible for other reasons (e.g., business). This left 586 eligible households, of which 571 were interviewed. A total of 15 known eligible households refused to participate, leaving a response rate for known eligibles of 97.6%. Of the 571 respondents, 231 households included children 2–6 years of age. Of these, 218 agreed to participate by providing samples (94.4% of those eligible), and urine samples were collected from 211 children and 213 adults. House dust was collected from 210 households, and of the households with vehicles (*n* = 214), vehicle dust was collected from 204.

For the final assessment, from a list of 1,375 households, 611 households were known to be eligible to participate. Of the remaining households, 486 had no farmworkers in the home, 125 houses were vacant, in 39 no adult was home after five visits, 92 had other reasons for not being eligible, and 22 households refused to participate. Of the 611 known eligible households, 595 participated for a response rate of 97.4%. Of the participating households, 236 had children 2–6 years of age in the household; of these, 207 agreed to participate in the sample collection for a response rate of 87.7%. Urine samples were collected from 202 adults and 204 children, and house and vehicle dust were collected from 203 and 177 households, respectively.

### Biomarker data

Table 1 shows the urinary metabolites by the limits of detection (LOD) for year 1 and year 4. As can be seen for the urinary metabolites, the most frequently measured metabolites were DMTP in both adult and child and to a lesser extent, DMDTP in both adult and child. Overall, 94% of adult samples in year 1 (baseline) and 93% in year 4 had detectable levels of DMTP compared with 88% and 84% respectively for children. For DMDTP, the percent detects were 55% in each of years 1 and 4 for adults and 45% and 28% respectively for children. The median concentration of the urinary metabolites were higher in year 4 than in year 1 for DMTP and DMDTP in adults and for DMTP for children. For DMP, the median concentration was below the LOD. The LOD for these metabolites varied between year 1 and year 4 because of changes in the analytical laboratory procedures. Thus, a direct comparison of the percentages of samples below the LOD is complicated.

### Dust data

The dust residue data showed azinphos-methyl having the highest percentage of detects in vehicles (86% and 84% in years 1 and 4, respectively) and in house dust (85% and 83% in years 1 and 4, respectively). Detection of phosmet was less pronounced in year 1 (21% in vehicles and 13% in house dust) than in year 4 (48% in vehicles and 45% in house dust). Malathion also showed an increase between years 1 and 4 with vehicle dust at 16% in year 1 and 39% in year 4, and house dust at 15% in year 1 and 26% in year 4. (Table 2) The median azinphos-methyl residues concentrations decreased in year 4; the others were all below the LOD. As with the urinary metabolites, the LOD varied between year 1 and year 4.

Table 3 reports the geometric mean concentrations and geometric standard deviations of the urinary metabolites DMP, DMTP, and DMDTP for adults and children by intervention and control group and by years 1 and 4 of the study. When we compared geometric mean values of adult DMP in year 1 and 4, the differences were not significant; neither were differences between intervention and control communities after adjusting for year, *p* = 0.26. For adult DMTP, there were significant increases within both the intervention and control communities between year 1 and year 4, *p* < 0.005; however, the differences were not significant between study communities after adjusting for year, *p* = 0.21. As with DMTP, DMDTP increased significantly in both intervention and control communities in year 4. In this case, the differences between intervention and control communities were marginally significant, with the intervention communities having higher mean concentrations after adjusting for year, *p* = 0.06. The same patterns held for the child urinary metabolites.

Table 4 shows pesticide residues in vehicle and house dust. When looking at vehicle dust, the mean azinphos-methyl residue concentrations decreased in both the intervention (although the decrease was nonsignificant, *p* = 0.11) and control communities in year 4 (where there was a significant decrease, *p* < 0.005) compared with year 1. Overall, however, there was no significant difference in decrease of mean azinphos-methyl concentrations between the intervention and control communities after adjusting for year, *p* = 0.16. When examining phosmet, its mean residue concentrations increased significantly in both intervention and control communities in year 4 compared with year 1; in addition, there was a marginally significant difference between intervention and control communities after adjusting for year, *p* = 0.07, with intervention communities having higher mean concentrations of phosmet. There was an increase in malathion in both intervention and control communities in year 4 compared with year 1; however, there was no significant difference between intervention and control communities after adjusting for year, *p* = 0.10.

For house dust, we saw a decrease in mean azinphos-methyl residue concentrations in both communities, but no significant differences between intervention and control communities after adjusting for year, *p* = 0.49. Mean phosmet residue concentrations increased in year 4, but there were no significant differences between communities after adjusting for year, *p* = 0.47. Finally mean concentrations of malathion residue in house dust decreased from year 1 to year 4 in both intervention and control communities; however, there were no significant differences between communities after adjusting for year, *p* = 0.43.

## Discussion

In this community randomized study of a community-based intervention to reduce pesticide exposure among farmworkers and their children, the findings show differences in urinary metabolites in the year 4 assessment compared with the year 1 assessment; however, for both adult and child DMPT and DMDTP, the mean metabolite concentrations increased in year 4. This was contrary to our hypothesis, in which we expected a decrease in our intervention communities. Although only adult and child DMDTP were marginally significantly different in the intervention compared with control communities, the differences were in an unexpected direction; that is, the intervention communities had higher mean concentrations of urinary metabolites. This result was also found in the dust residue data. This is a disappointing outcome. We examine a number of reasons why these findings may have resulted.

Two rulings by the U.S. EPA may have contributed to our findings. First, the U.S. EPA increased the re-entry interval for azinphos-methyl from 3 days to 14 days during the project (U.S. EPA 1999, 2002). A related U.S. EPA ruling was that azinphos-methyl, because of its high toxicity, should be phased out by 2011(U.S. EPA 2002). These rulings may have led to use of other pesticides and could have contributed to a shift in the urinary metabolite and dust residue profiles. Indeed, azinphos-methyl decreased between our two assessment intervals, whereas phosmet increased. Phosmet, which metabolizes similarly to azinphos-methyl, did not have an increase in the prescribed re-entry period. That is, farmworkers continued to enter a phosmet-applied field at a 3-day interval rather than 14 days. This may have resulted in an excess of phosmet compared with azinphos-methyl. Data from the Washington State Agricultural Chemical Use Surveys (U.S. Department of Agriculture 2000) show phosmet use increasing throughout the state during the period of this study. This might help explain why the amounts of urinary metabolites showed an increase.

Another potential explanatory factor is that applications of pesticides vary by year and depend on many things including weather, prevalence of pests, and farming practices. What we report could partially be an increased use of pesticides due to a variety of factors. We examined the Washington State Agricultural Chemical Use Surveys (U.S. Department of Agriculture 2002b) to identify differences in pesticide usage in the years in question; however, the usage data are collected biennially, and they were not collected during the year the final assessment (year 4) was made. It is unlikely that growers change spray patterns drastically in the absence of catastrophic changes; however, smaller changes in climate or pests may result in slight differences in pesticide use. It is difficult to assess these small differences because the half-life of OPs in the body is very short. In terms of dust, there should have been more stability because the residues last longer in the home where they are not degraded by the weather. It would have been desirable to assess pesticide usage patterns during the life of the project; unfortunately, available resources did not allow us to take pesticide usage patterns into account when designing the project.

Another potential explanation is that the timing of pesticide spray varies by time of the year. There is the possibility that either year 1 or year 4 was particularly different for pesticide application. For example, thinning, the removal of buds while flowering, is a time of heavy pesticide usage. During the follow-up survey, spraying may have continued because of a late spring during the data collection period; thus, we may have picked up more pesticides then. Again, we did not have the resources to ascertain the spraying patterns of each of the growers during the study.

The reliability of measurements across different analytic methods and laboratories has presented an ongoing challenge for those interested in interpreting results across studies. We found, for example, that the LOD of pesticides varied from baseline to follow-up. Regardless, however, for this study, the same laboratory conducted the analyses in both years. In addition, the University of Washington Environmental Health Laboratory competed successfully in an extensive testing of dialkylphosphate metabolite analysis that included the Centers for Disease Control and Prevention. We therefore concluded that the results produced from the year 1 and year 4 were very comparable despite the differences in the LOD.

Barr and others have shown that individuals of different ages metabolize pesticides differently, and certain racial and ethnic groups have genetic susceptibilities that influence the rate at which pesticides are metabolized (Barr et al. 2006). Children, for example, are thought to metabolize pesticides more slowly than adults. Adults may have genetic predispositions to be faster or slower metabolizers of pesticides. The natural variability in metabolic efficiencies may have diluted our ability to detect cross-sectional changes over time.

Another potential weakness is that we used cross-sectional assessment to detect differences in our study groups. There are three major reasons we elected to follow a cross-sectional design. First, the farmworkers in the valley tend to be quite mobile, moving within the valley and to other parts of the United States. It would have been labor- and resource-intensive to follow a cohort, and we did not have the resources to do that. Second, with this community study, we hoped to change community norms and practices around pesticides. Community-wide changes were thought to be detectable in different cross-sectional samples. Finally, many Hispanic farmworkers in the valley are undocumented. It was important to us to obtain a representative sample of farmworkers; thus, we decided to reduce the chances of noncompliance with the survey by not asking about documentation status. Our CAB further advised us not to ask people for their full names because it might intimidate undocumented workers. Thus, we used a household randomization scheme, rather than individual. We rostered farmworkers in a household by their first names and birthdays, and selected the participant based on birthdate. The embedded cohort, calculated by first name and birthdate, was too small for robust analysis.

Some have argued that the precision of a cohort is outweighed by attrition and bias (Commit Research Group 1995). Nevertheless, a cross-sectional design also has limitations, especially where differences are expected to be small. The outcome assessment sample may not have received sufficient dose of the intervention to lead to behavior change. Alternatively, changes in urinary metabolites may not be the best way to measure behavior change. Subsequent analyses will address behavioral changes as reported by the farmworkers participating in the study.

Some study design issues may have contributed to our null findings. First is the issue of communities selected and contamination. Although the communities we selected for participation have some geographic distance from each other, the overall area included is relatively small. Equally important, the farmworkers travel around the valley working in the crops, so it was difficult to be sure that farmworkers in the control communities did not receive some aspects of the intervention. There may have been contamination between the intervention and control communities. The process evaluation conducted for this study indicates that there was some contamination in that up to 20% of farmworkers in control communities also participated in intervention activities. A full description of the process evaluation is under preparation.

Few studies have emphasized things farmworkers and their families can do to avoid pesticide residues in the home, relying instead on actions farmworkers can take at work to reduce their own exposure. Projects of this type have included facilitating exposure monitoring (Davies et al. 1982; Fenske et al. 1990; Nigg et al. 1986), personal protective equipment monitoring (Davies et al. 1982; Fenske et al. 1990; Gomes et al. 1999; McCauley et al. 2003; Nigg et al. 1986; Putnam and Bennett 1983; van der Jagt et al. 2004), pesticide handlers interventions (Davies et al. 1982; Nigg et al. 1986; Putnam and Bennett 1983), and biological monitoring interventions (Fillmore and Lessenger 1993). Few studies have examined the take-home pathway and what can be done to interrupt that pathway. Some studies have recently begun to focus on the home (McCauley et al. 2006a; Williams et al. 2006) as a way to protect farmworkers and their families. To our knowledge, this is the first randomized community trial to incorporate both work and home protective practices.

This study was novel in that it used a community randomized study to interrupt the take-home pathway. The dose of intervention in community trials is spread across a wide population base. This makes it difficult to detect small differences that can be seen in a sample of individuals from the community. A more intensive one-on-one intervention may be more successful than a community-wide approach. Certainly there is ample evidence that one-on-one interventions leads to behavior change in reducing pesticide exposure (Sklansky et al. 2003; Vela-Acosta et al. 2002), although community-wide changes are harder to detect.

Alternatively, the intervention simply may not have been effective. We may have conducted the wrong type of activities to reach our targeted population. Although this may not be totally dismissed, participation in our intervention activities suggests our activities were agreeable to the farmworkers in the valley. Further, focus groups indicate that the intervention activities were appropriate for the population. Finally, our CAB recommended the activities, indicating they were culturally appropriate for farmworkers.

## Conclusions

In this community randomized trial designed to reduce the take-home pathway of pesticides among children of farmworkers, we found no significant decreases in urinary pesticide metabolite concentrations or in pesticide residue concentrations in house and vehicle dust from intervention community households compared with control community households after adjusting for baseline. We used a community organization model that involved community members in all aspects of the intervention. The intervention was comprehensive and appeared to be agreeable to and suitable for farmworkers in the Yakima Valley. Contrary to our hypotheses, we found more exposure at the end of the study than at the beginning. More analysis is needed to identify ways to interrupt the take-home pathway of pesticide exposure.

## Figures and Tables

**Table 1:** Percentage of child and adult urine samples containing detectable concentrations of DMP, DMTP, and DMDTP and the limits of detection (LOD) for baseline (year 1) and follow-up (year 4).

				Quantiles (μg/L)					
	Year	No.	Percent detectable	Min	25th	50th	75th	95th	Max
Adult									
DMP	1	205	17.1	< LOD	< LOD	< LOD	< LOD	31.8	102.4
	4	202	29.2	< LOD	< LOD	< LOD	8.1	71.9	176.3
DMTP	1	205	93.7	< LOD	3.3	9.7	29.6	169.9	1960.0
	4	202	92.6	< LOD	15.2	56.2	227.2	1243.6	4177.6
DMDTP	1	205	54.6	< LOD	< LOD	1.2	4.0	21.9	185.0
	4	202	55.0	< LOD	< LOD	6.0	21.4	148.0	2112.4
Child									
DMP	1	204	18.1	< LOD	< LOD	< LOD	< LOD	18.0	41.4
	4	204	21.7	< LOD	< LOD	< LOD	< LOD	9.9	65.9
DMTP	1	204	87.7	< LOD	2.3	5.7	12.2	37.0	136.0
	4	204	84.3	< LOD	9.8	21.5	41.0	181.8	734.4
DMDTP	1	204	44.6	< LOD	< LOD	< LOD	2.9	10.2	38.6
	4	204	28.4	< LOD	< LOD	< LOD	6.0	29.4	258.9

Abbreviations: Max, maximum; Min, minimum. LOD in year 1 were as follows: DMP, 7.2, DMTP, 1.1, and DMDTP, 0.65 μg/L; in year 4 the LOD were 5.0 μg/L.

**Table 2:** Percentage of vehicle and house dust samples containing detectable concentrations of azinphos-methyl, phosmet, and malathion and the LOD for baseline (year 1) and follow-up (year 4).

				Quantiles (ng/L)					
	Year	No.	Percent detectable	Min	25th	50th	75th	95th	Max
Vehicle									
Azinphos-methyl	1	183	86.4	< LOD	203	709	2,321	9,530	38,300
	4	172	84.3	< LOD	126	367	1,400	4,325	17,800
Phosmet	1	183	20.8	< LOD	< LOD	< LOD	< LOD	857	10,540
	4	172	47.7	< LOD	< LOD	< LOD	218	2,219	10,600
Malathion	1	183	15.8	< LOD	< LOD	< LOD	< LOD	290	2,120
	4	172	39.0	< LOD	< LOD	< LOD	122	714	21,504
House									
Azinphos-methyl	1	149	84.6	< LOD	200	483	1,305	4,907	10,127
	4	179	83.4	< LOD	110	284	805	2,541	10,487
Phosmet	1	149	12.8	< LOD	< LOD	< LOD	< LOD	704	3,743
	4	179	44.7	< LOD	< LOD	< LOD	207	1,632	13,804
Malathion	1	149	15.4	< LOD	< LOD	< LOD	< LOD	284	1,030
	4	179	25.7	< LOD	< LOD	< LOD	70	244	767

Abbreviations: Max, maximum; Min, minimum. LOD in year 1 were as follows: azinphosmethyl 80, phosmet 110, and malathion 80 ng/g; in year 4 the LOD for all were 70 ng/g.

**Table 3:** Comparison of the geometric means of the urinary metabolite concentrations for the intervention and control communities at baseline (year 1) and follow-up (year 4).

	Year	GM (μg/L)	GSD	Fold difference (year 4/year 1)	*p*-Value	Fold difference int (year 4/year 1)/ctl (year 4/year 1)	*p*-Value
Adult DMP						0.6	0.26
Intervention	1	1.7	7.1	1.2	0.33		
	4	2.1	9.0				
Control	1	0.9	7.5	2.0	0.11		
	4	1.8	7.0				
Adult DMTP						1.3	0.21
Intervention	1	12.3	4.9	6.6	< 0.005		
	4	81.4	6.0				
Control	1	9.7	4.9	5.0	< 0.005		
	4	48.3	3.1				
Adult DMDTP						2.0	0.06
Intervention	1	0.9	8.8	8.8	< 0.005		
	4	8.1	6.6				
Control	1	1.1	5.7	4.4	< 0.005		
	4	5.0	7.9				
Child DMP						1.3	0.29
Intervention	1	2.8	2.8	1.3	0.23		
	4	3.4	1.9				
Control	1	2.6	3.4	0.9	0.43		
	4	2.5	2.8				
Child DMTP						1.1	0.35
Intervention	1	4.5	3.4	4.0	< 0.005		
	4	18.2	4.3				
Control	1	6.1	3.8	3.6	< 0.005		
	4	21.9	3.1				
Child DMDTP						1.8	0.07
Intervention	1	0.8	4.4	4.1	< 0.005		
	4	3.2	3.9				
Control	1	1.0	5.1	2.3	< 0.005		
	4	2.2	4.2				

Abbreviations: ctl, control; GM, geometric mean; GSD, geometric standard deviation; int, intervention.

**Table 4:** Comparison of the geometric means of the dust pesticide residue concentrations for the intervention and control communities at baseline (year 1) and follow-up (year 4).

	Year	GM (μg/L)	GSD	Fold difference (year 4/year 1)	*p*-Value	Fold difference int (year 4/year 1)/ctl (year 4/year 1)	*p*-Value
Vehicle azinphos-methyl						1.4	0.16
Intervention	1	635.3	5.5	0.7	0.11		
	4	463.9	4.9				
Control	1	713.3	6.0	0.5	< 0.005		
	4	368.0	5.1				
Vehicle phosmet						3.6	0.07
Intervention	1	7.4	24.2	9.6	< 0.005		
	4	69.9	7.8				
Control	1	25.6	7.1	2.7	0.01		
	4	67.7	7.2				
Vehicle malathion						0.4	0.10
Intervention	1	23.2	5.5	2.1	0.05		
	4	47.4	6.2				
Control	1	7.0	7.1	5.5	< 0.005		
	4	38.8	5.1				
House azinphos-methyl						1.0	0.49
Intervention	1	558.6	4.5	0.6	0.01		
	4	324.2	4.3				
Control	1	500.8	4.0	0.6	< 0.005		
	4	292.6	3.8				
House phosmet						0.9	0.47
Intervention	1	21.2	13.7	3.8	0.01		
	4	80.0	5.4				
Control	1	10.1	9.4	4.1	0.03		
	4	42.2	10.7				
House malathion						1.1	0.43
Intervention	1	58.7	3.1	0.6	0.12		
	4	35.0	4.2				
Control	1	43.9	3.6	0.5	0.11		
	4	23.2	4.5				

Abbreviations: ctl, control; GM, geometric mean; GSD, geometric standard deviation; int, intervention.
